# Isolation and Characterization of Microsatellite Markers and Analysis of Genetic Diversity in Chinese Jujube (*Ziziphus jujuba* Mill.)

**DOI:** 10.1371/journal.pone.0099842

**Published:** 2014-06-16

**Authors:** Siqi Wang, Ying Liu, Liying Ma, Huabo Liu, Yan Tang, Liping Wu, Zhe Wang, Yingyue Li, Rongling Wu, Xiaoming Pang

**Affiliations:** National Engineering Laboratory for Tree Breeding, Key Laboratory of Genetics and Breeding in Forest Trees and Ornamental Plants, Ministry of Education, Center for Computational Biology, College of Biological Sciences and Biotechnology, Beijing Forestry University, Beijing, China; Key Laboratory of Horticultural Plant Biology (MOE), China

## Abstract

Chinese jujube (*Ziziphus jujuba* Mill, 2n = 2× = 24, Rhamnaceae) is an economically important Chinese native species. It has high nutritional value, and its medicinal properties have led to extensive use in traditional oriental medicine. The characterization of genotypes using molecular markers is important for genetic studies and plant breeding. However, few simple sequence repeat (SSR) markers are available for this species. In this study, 1,488 unique SSR clones were isolated from *Z. jujuba* ‘Dongzao’ using enriched genomic libraries coupled with a three-primer colony PCR screening strategy, yielding a high enrichment rate of 73.3%. Finally, 1,188 (80.87%) primer pairs were amplified successfully in the size expected for ‘Dongzao’. A total of 350 primer pairs were further selected and evaluated for their ability to detect polymorphisms across a panel of six diverse cultivars; among these, 301 primer pairs detected polymorphisms, and the polymorphism information content (PIC) value across all loci ranged from 0.15 to 0.82, with an average of 0.52. An analysis of 76 major cultivars employed in Chinese jujube production using 31 primer pairs revealed comparatively high genetic diversity among these cultivars. Within-population differences among individuals accounted for 98.2% of the observed genetic variation. Neighbor-joining clustering divided the cultivars into three main groups, none of which correspond to major geographic regions, suggesting that the genetics and geographical origin of modern Chinese jujube cultivars might not be linked. The current work firstly reports the large-scale development of Chinese jujube SSR markers. The development of these markers and their polymorphic information represent a significant improvement in the available Chinese jujube genomic resources and will facilitate both genetic and breeding applications, further accelerating the development of new cultivars.

## Introduction

Chinese jujube (*Ziziphus jujuba* Mill, 2n = 2× = 24, Rhamnaceae) is an economically important Chinese native species. Chinese jujube has been cultivated for at least 3,000 years, and archaeological evidence indicates that it was utilized 7,700 years ago in China [1]. Its fruit has a high nutritional value because it contains high levels of vitamin C, abundant phenolic compounds, high carbohydrate and mineral (particularly potassium and iron) content, and the highest level of cyclic AMP among higher plants [2], [3]. It can be consumed as fresh, dried or processed fruit. In addition, the fruit has interesting medicinal properties and has been extensively used in traditional oriental medicine for its analeptic, palliative and antibechic purposes [4], [5].

Chinese jujube is well adapted to various climate and soil conditions (pH 5.5–8.5); however, well drained, sandy or loamy soils combined with high levels of sunlight produce high yield and fruit quality [1]. Chinese jujube is distributed throughout China except for the most northern part, i.e., Heilongjiang province. The total growing area is estimated at more than 1.5 million hectares, and average annual production amounts to 3.5 million tons (dried weight) [6]. The top 6 provinces ranked in order of production are Xinjiang, Shannxi, Shanxi, Hebei, Shandong and Henan; together, these provinces account for 90% of the entire yield. This plant has been introduced to more than 30 countries, among which only South Korea has engaged in commercial production.

During the long-term process of natural evolution and artificial selection, the Chinese jujube has developed a wide range of variation, and more than 800 cultivars have been reported [7]. These cultivars are distributed throughout China and are propagated vegetatively either by grafting onto rootstock or as rooted cuttings [8]; the origin of most accessions is obscure because of the frequent exchange of plant material among different cultivation areas, and the lack of cultivar history documentation. The naming of jujube cultivars and types is confusing, particularly the use of homonyms and synonyms. For example, there are more than 20 different local names for the cultivar ‘Dongzao,’ a cultivar that provides the best fresh fruit quality. Therefore, there is an urgent need for accurate germplasm and cultivar identification.

Previously, the classification of cultivars had been based mainly on morphological characteristics and their usages [1]. However, traditional morphological identification has a number of limitations, including low polymorphism, low heritability, late expression and vulnerability to environmental influences [9]. The advent of molecular markers offers a promising tool for Chinese jujube cultivar identification. Several molecular markers, such as random amplified polymorphic DNA (RAPD), amplified fragment length polymorphisms (AFLPs) and sequence-related amplified polymorphisms (SRAPs), have been used to differentiate various Chinese jujube cultivars [10], [11]. However, the use of these marker systems is laborious and produces complex patterns that are inconvenient for database building [12]. SSR, also known as microsatellite DNA, is widely distributed in eukaryotic genomes [13]. Due to their abundance, high polymorphism, codominance, stability and suitability for automated analysis, SSR markers are widely used in germplasm conservation, genetic diversity analysis, the study of genetic relationships, genetic mapping, DNA fingerprinting and molecular marker-assisted breeding [14].

Furthermore, the genetics of Chinese jujube lags behind that of many other fruit crops mainly due to a lack of both segregating populations and efficient markers. Because the jujube has a small flower (which is difficult to work with) and a low rate of kernel in the seed, no successful artificial controlled crossing has yet been attempted to obtain structured pedigrees with sufficient offspring. Lu et al. [15] developed the only F_1_ segregating population in Chinese jujube using an AFLP marker to identify full sib seedlings from open pollinated seedlings. These researchers subsequently constructed the first genetic map of jujube using AFLP markers [16]; however, no co-dominant marker was integrated into the map, limiting its further utilization. As proposed in the ‘breeding without breeding’ (BWB) method, molecular markers such as SSRs can be employed to obtain structured pedigrees from open pollinated progenies [17]. Thus, the development of SSR markers will also benefit the development of more structured pedigrees for the breeding and genetic analysis of important traits for outcrossing Chinese jujube.

Several strategies are available for identifying new SSRs from a genome [18]. Ma et al. [19] developed 25 SSR primers using the selectively amplified microsatellite (SAM) approach. EST SSRs can be mined from expressed sequence tags (ESTs) and cDNAs [20]. Recently, Liu et al. [21] developed 119 EST-SSRs from a Chinese jujube fruit cDNA library. However, the number of available markers remains insufficient for genetic and breeding research of Chinese jujube. Therefore, additional SSR markers must be developed for genetic studies of important traits in Chinese jujube, such as fruit quality and disease resistance. Unfortunately, publicly available DNA sequence data are limited for Chinese jujube. The use of traditional SSR-enriched libraries remains one of the methods of choice for the development of large-scale SSR markers for less-studied species [18], [22].

In this study, we report the identification and characterization of 1,118 unique SSR markers developed from SSR-enriched genomic libraries of Chinese jujube. A set of 350 SSR markers was evaluated for their ability to detect polymorphisms across a panel of six diverse cultivars. Since the genetic base of the main cultivars currently employed in the production of Chinese jujube in China is still not clear, a total of 31 polymorphic primer pairs were utilized to determine the range of genetic diversity among them and analyze their genetic relationships.

## Results

### SSR isolation by SSR-enriched libraries

Six SSR-enriched DNA libraries were constructed using a combination of three restriction enzymes and two types of probes; from these libraries, 6,720 colonies were screened using the three-primer method [23], as shown in Table 1. In total, 2,030 positive colonies were obtained. After sequencing, 1,854 (91.3%) sequences were found to harbor SSR loci, confirming the efficiency of the three-primer strategy. After eliminating redundant sequences, 1,488 unique SSR clones remained, corresponding to an enrichment rate of 73.3% across all libraries. A set of 368 clones was excluded because the sequences flanking the SSR motifs were too short to design both forward and reverse primers. Finally, 1,120 unique SSR clones were identified and used to design primers. The enzyme *Hae*III produced the highest average percentage of efficient clones (61.5%); *Alu*I (57.6%) produced the second highest, and *Rsa*I (46.8%) produced the least. No difference in the efficiency of the two probes, (AC)_15_ and (AG)_15_, was observed.

**Table 1:** The efficacy of SSR isolation from six SSR-enriched DNA libraries.

Library	Motif	clones sequenced	SSR clones	Efficient SSR clones
ALUAG	GA/TC	322	300	187
RSAAG	GA/TC	328	302	161
HAEAG	GA/TC	338	309	201
ALUAC	CA/TG	391	353	223
RSAAC	CA/TG	346	312	154
HAEAC	CA/TG	305	278	194
Total		2030	1854	1120

### Characteristics and distribution of SSRs

Among the unique SSR clones, 2,128 SSR loci were identified; 673 clones contained more than one SSR loci and 258 clones were present in compound formation. Among the clones obtained, 975 and 1,153 loci were discovered using the probes (AC)_15_ and (AG)_15_, respectively. The distribution of SSR motifs across different libraries is summarized in Table 2. Of the 2,128 identified SSR loci, 1,691 were dinucleotides (79.46%), 145 were trinucleotides (6.81%), and 292 were motifs larger than three nucleotides (13.72%). As shown in Table 3, the predominant motif was the expected type in all six libraries, i.e., AG/CT for ALUAG (64.81%), RSAAG (61.48%) and HAEAG (58.70%) and AC/GT for ALUAC (42.51%), RSAAC (47.04%) and HAEAC (45.06%).

**Table 2:** Distribution of different repeat types in six SSR-enriched DNA libraries.

Unit size	Number of SSRs						Total
	ALUAG	RSAAG	HAEAG	ALUAC	RSAAC	HAEAC	
2	253	223	272	371	251	321	1691 (79.46%)
3	30	20	41	23	13	18	145 (6.81%)
4	25	29	33	43	31	39	200 (9.40%)
5	7	7	9	7	7	5	42 (1.97%)
6	9	4	13	10	2	12	50 (2.35%)
Total	324	283	368	454	304	395	2128

**Table 3:** Frequencies of motif types in six SSR-enriched DNA libraries.

Unit size	Number of SSRs						Total
	ALUAG	RSAAG	HAEAG	ALUAC	RSAAC	HAEAC	
Dinucleotide motifs							
AC/GT	19	34	22	193	143	178	589
AG/CT	210	174	216	77	63	63	803
AT/AT	24	15	34	101	44	80	298
CG/CG					1		1
Trinucleotide motifs							
AAC/GTT	2	2	2		3	3	12
AAG/CTT	14	4	19	9	5	3	54
AAT/ATT	9	9	9	12	5	11	55
ACC/GGT	2	2	1	1			6
ACG/CGT			1				1
ACT/AGT	1	1	1			1	4
AGC/CTG	1		3	1			5
AGG/CCT			2				2
ATC/ATG	1	2	3				6
Tetranucleotide motifs	25	29	33	43	31	39	200
Pentanucleotide motifs	7	7	9	7	7	5	42
Hexanucleotide motifs	9	4	13	10	2	12	50
Total	324	283	368	454	304	395	2128

### Primer design and evaluation

From the 2,128 identified loci, 1,469 primer pairs were designed and validated for ‘Dongzao’. Finally, 1,188 (80.87%) primer pairs successfully amplified products of the expected size (Table S1). Among these primer pairs, 1,039 (87.5%) yielded the single motifs, and the remaining 149 (12.5%) generated the complex motifs. The markers that were successfully amplified had repeat numbers ranging from 3 to 37. A set of 350 primer pairs with the clearest banding patterns was selected to evaluate polymorphisms in six Chinese jujube cultivars (Table S2). Of the 350 primer pairs, 301 detected polymorphisms. The polymorphism information content (PIC) values ranged from 0.15 to 0.82 (Table S2), with an average of 0.52; 157 primer pairs had PIC values greater than 0.50. Among the 301 polymorphic primer pairs, 270 contained dinucleotide repeats exhibiting a mean PIC value of 0.52, 12 contained trinucleotide repeats exhibiting a mean PIC value of 0.46, and the remaining 19 pairs with repeats longer than three nucleotides exhibited a mean PIC value of 0.50. The correlation coefficient between the PIC and SSR length was 0.12 (p = 0.0206).

### Genetic diversity analysis of the main Chinese jujube cultivars

To elucidate the genetic diversity of the cultivars used in Chinese jujube production, we analyzed a set of the 76 major cultivars using 31 SSR loci. A total of 178 SSR marker alleles were detected, with an average of 5.7 alleles per locus (Table 4). The BFU0308 primer pair produced the highest number (15) of alleles, whereas BFU0584 and BFU0614 produced the lowest number (2 alleles). The effective number of alleles per locus, which reflects the evenness of allelic frequencies, varied from 1.314 to 7.415, with an average of 3.148. The observed heterozygosity (H_o_) ranged from 0.250 to 1.000, with an average of 0.678. The expected heterozygosity (H_e_) ranged from 0.239 to 0.865, with an average of 0.621. The PIC value ranged from 0.229 for BFU0521 to 0.851 for BFU0308. The overall fixation index (*F*) was −0.081, indicating a slight excess of heterozygotes.

**Table 4:** Characterization of 31 SSR loci in 76 Chinese jujube cultivars.

Locus	Repeat motif	*Na*	*Ne*	Ho	He	*F*
BFU0277	(GA)11	6	2.824	0.566	0.646	0.124
BFU0083	(CT)13	5	4.339	0.827	0.770	−0.074
BFU0574	(CA)7	10	4.518	0.882	0.779	−0.132
BFU1205	(CA)8	5	4.823	0.855	0.793	−0.079
BFU0528	(TC)8	8	5.548	0.878	0.820	−0.072
BFU1157	(GA)9	9	4.777	0.853	0.791	−0.079
BFU0581	(CA)7	11	4.666	0.921	0.786	−0.172
BFU1248	(ATTA)4	5	3.212	0.789	0.689	−0.146
BFU0377	(CT)10	12	4.044	0.819	0.753	−0.089
BFU0561	(CT)7	9	3.762	0.882	0.734	−0.201
BFU1383	(ATTT)3	3	2.713	0.667	0.631	−0.056
BFU0308	(TC)11	15	7.415	1.000	0.865	−0.156
BFU0249	(GT)12	4	1.996	0.434	0.499	0.130
BFU0263	(CT)11	4	2.331	0.750	0.571	−0.313
BFU0286	(AG)10	5	2.902	0.737	0.655	−0.124
BFU0467	(TC)9	9	3.954	0.893	0.747	−0.196
BFU0473	(AG)9	4	1.659	0.382	0.397	0.039
BFU0478	(TC)9	3	1.371	0.253	0.271	0.065
BFU0479	(TC)9	4	2.317	0.159	0.568	0.720
BFU0501	(AG)8	3	1.829	0.474	0.453	−0.045
BFU0521	(TC)8	4	1.314	0.250	0.239	−0.046
BFU0539	(TC)8	5	2.433	0.684	0.589	−0.162
BFU0564	(TC)7	3	2.433	0.840	0.589	−0.426
BFU0584	(CT)7	2	1.583	0.432	0.368	−0.175
BFU0586	(TC)7	4	2.849	0.724	0.649	−0.115
BFU1178	(TG)9	4	3.075	0.680	0.675	−0.008
BFU1279	(TTAA)4	5	2.541	0.658	0.606	−0.084
BFU1409	(CA)6	7	3.409	0.907	0.707	−0.283
BFU0580	(TC)7	5	2.433	0.684	0.589	−0.162
BFU0614	(CT)6	2	1.532	0.342	0.347	0.015
BFU0733	(CT)9	3	2.990	0.800	0.666	−0.202
Mean		5.7	3.148	0.678	0.621	−0.081

*Na*: Allele number; *Ne*: effective allele number; Ho: Observed heterozygosity; He: Expected heterozygosity; *F*: Fixation index

Based on their geographical distribution, 76 cultivars were divided into seven populations (Pop1–Pop7) (Table 5). The number of alleles per locus ranged from 3.097 in Pop1 to 4.548 in Pop6. Private SSR alleles were present in all populations, with the highest numbers occurring in Pop4 (4), Pop5 (6) and Pop6 (7)(Table 6). AMOVA analysis revealed that a very low percentage of variation was partitioned among the populations (Table 7). Of the total genetic variance, 99.80% was ascribed to differences within populations.

**Table 5:** 76 Chinese jujube cultivars used in the study.

Pop	Cultivar name	Orgin	Uses	Pop	Cultivar name	Orgin	Uses
1	Gagazao	Beijing	Fresh	5	Chuanling	Shandong	Dried
1	Beijingmaya	Beijing	Fresh or dried	5	Huluchanghong	Shandong	Dried
1	Chaoyangyuanzao	Liaoning	Fresh	5	Duanguochanghong	Shandong	Dried
2	Linzexiaozao	Gansu	Dried	5	Yuanlingzao	Shandong	Dried
2	Gansuxiaokou	Gansu	Dried	5	Yuanlizao	Shandong	Fresh
2	Minqinxiaozao	Gansu	Fresh or dried	5	Shandonglizao	Shandong	Fresh
2	Dunhuangdazao	Gansu	Fresh or dried	5	Xuezao	Shandong	Fresh
2	Shaanxigedazao	Shaanxi	Dried	5	Lizao	Shandong	Fresh
2	Shanxijidan	Shaanxi	Fresh	5	Chengwudongzao	Shandong	Fresh
2	Bashenghu	Shaanxi	Fresh or dried	5	Dabailing	Shandong	Fresh
2	Goutouzao	Shaanxi	Fresh or dried	5	Kongfusucui	Shandong	Fresh
3	Yuanzao	Anhui	Fresh	5	Damaya	Shandong	Fresh or dried
3	Lianxianmuzao	Guangdong	Candied	5	Jinsi4	Shandong	Fresh or dried
3	Suizhoudazao	Hubei	Candied	6	Muzao	Shanxi	Dried
3	Xupujidan	Hunan	Fresh	6	Guantanzao	Shanxi	Dried
3	Zhongqiusucui	Hunan	Fresh	6	Xiangzao45	Shanxi	Dried
3	Yiwuzao	Zhejiang	Candied	6	Muzaokanglie	Shanxi	Dried
3	Nanjingzao	Zhejiang	Candied	6	Zhongyangmuzao	Shanxi	Dried
3	Shengxianbaipu	Zhejiang	Candied	6	Xiangzao10	Shanxi	Dried
4	Popozao	Hebei	Dried	6	Jianzao	Shanxi	Fresh or dried
4	Popozao52	Hebei	Dried	6	Lichengxiaozao	Shanxi	Fresh or dried
4	Lajiaozao	Hebei	Fresh	6	Jishanbanzao	Shanxi	Fresh or dried
4	Jidanzao	Hebei	Fresh	6	Linfenmizao	Shanxi	Fresh or dried
4	Dongzao6	Hebei	Fresh	6	Hupingzao	Shanxi	Fresh or dried
4	Chengtuozao	Hebei	Fresh	6	Pingshunjunzao	Shanxi	Fresh or dried
4	Dongzao38	Hebei	Fresh	6	Junzao	Shanxi	Fresh or dried
4	Dongzao100	Hebei	Fresh	6	Banzao	Shanxi	Fresh or dried
4	Dongzao70	Hebei	Fresh	7	Guangyangdazao	Henan	Fresh or dried
4	Zaocuiwang	Hebei	Fresh	7	Lingbaodazao	Henan	Fresh or dried
4	Wuhezao	Hebei	Fresh or dried	7	Changjixinzao	Henan	Dried
4	Maya	Hebei	Fresh or dried	7	Changjixinzao10	Henan	Dried
4	Jinsixiaozao	Hebei	Fresh or dried	7	Manmanzao	Henan	Fresh
4	Zanhuangdazao	Hebei	Fresh or dried	7	Wutouzao	Henan	Fresh
4	Zizao	Hebei	Fresh or dried	7	Liuyuexian	Henan	Fresh
4	Chuangan	Hebei	Fresh or dried	7	Dayewuhe	Henan	Fresh
4	Wuhezao72	Hebei	Fresh or dried	7	Mayizao	Henan	Fresh
4	Xiaozao	Hebei	Fresh or dried	7	Huizao154	Henan	Fresh or dried
5	Yuanlingxiaozao	Shandong	Dried	7	Huizao3	Henan	Fresh or dried

**Table 6:** Summary statistics of genetic variation at 31 SSR loci in within the *Ziziphus jujuba* populations.

Pop	*N*	*Na*	*Ne*	*Ho*	*He*	*F*	*Pa*
**Pop1**	3	3.097	2.727	0.785	0.588	−0.336	1
**Pop2**	8	3.581	2.679	0.631	0.575	−0.088	3
**Pop3**	8	3.581	2.641	0.641	0.551	−0.150	3
**Pop4**	18	4.484	2.988	0.695	0.606	−0.128	4
**Pop5**	14	4.387	3.036	0.698	0.612	−0.131	6
**Pop6**	14	4.548	2.883	0.689	0.601	−0.139	7
**Pop7**	11	4.161	2.869	0.638	0.588	−0.080	3
**mean**	10.857	3.977	2.832	0.683	0.589	−0.150	4

*Na*: Allele number; *Ne*: effective allele number; Ho: Observed heterozygosity; He: Expected heterozygosity; *F*: Fixation index; *Pa*: Private alleles

**Table 7:** Analysis of molecular variance (AMOVA) among 76 Chinese jujube cultivars from seven geographical populations, based on 31 SSR markers.

Source of variation	d.f.	Sum of squares	Variance components	Percentage of variation
Among populations	6	55.528	0.01815	0.20
Within populations	145	1286.491	8.87235	99.80
Total	151	1342.020	8.89051	

### Genetic relationships among the main Chinese jujube cultivars

**Figure 1:**
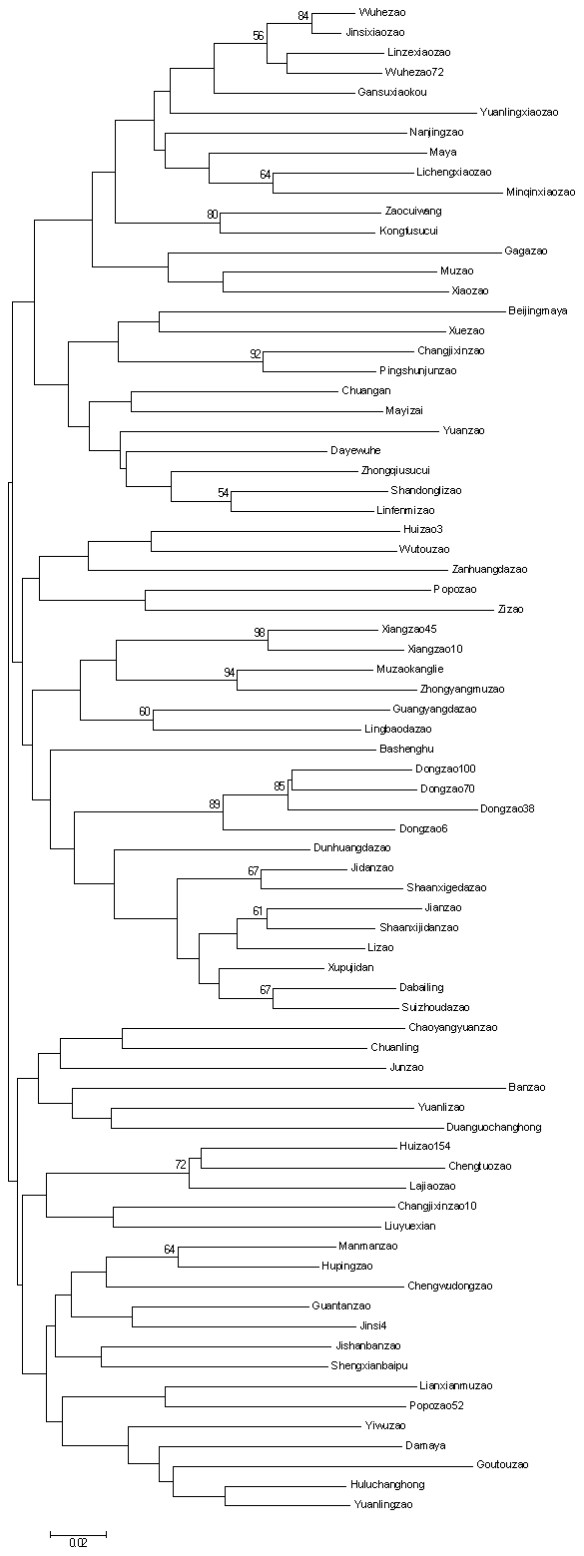
Dendrogram of the genetic relationships among 76 Chinese jujube cultivars based on SSR polymorphism. The dendrogram was generated using a simple matching coefficient based on 31 polymorphic primer pairs. Cluster analysis was performed using the neighbor-joining method. Bootstrap values obtained from 1000 replicate analyses higher than 50% are indicated on the nodes.

The simple matching pairwise distances ranged from 0.027 (‘Wuhezao’ and ‘Jinsixiaozao’) to 0.429 (‘Huizao154’ and ‘Beijingmaya’) among the 76 cultivars, with an overall mean of 0.269. The distances did not differ greatly between populations (0.263±0.005). The NJ tree based on SSR data grouped the 76 cultivars into three main groups (Figure 1). All three groups contained cultivars from seven populations except that the second group includes no cultivar from Pop1. The first group comprised 26 cultivars, including the ‘Jidanzao’ cultivar series (‘Jidanzao’ and ‘Shanxijidanzao’) and the ‘Xiaozao’ cultivar series (‘Jinsixiaozao,’ ‘Minqinxiaozao,’ ‘Lichengxiaozao,’ ‘Yuanlingxiaozao,’ ‘Gansuxiaokou’ and ‘Linzexiaozao’); The second group comprised 25 cultivars, including the ‘Dongzao’ cultivar series (‘Dongzao6,’ ‘Dongzao100,’ ‘Dongzao38,’ ‘Dongzao70’ and ‘Chengwudongzao’) and the ‘Xiangzao’ cultivar series (‘Xiangzao45’ and ‘Xiangzao10’). The third group contained 25 cultivars.

## Discussion

### Genomic SSR marker development efficiency

Various types of molecular markers have been developed since the advent of RFLP technology; SSRs and single nucleotide polymorphisms (SNPs) have been the principal markers utilized in plant genetic analysis and marker-assisted breeding [24], [25]. However, it is costly to develop SNPs for a less-studied plant such as Chinese jujube, for which only a few SSR markers have been reported [19]. Therefore, the development of a large number of SSR markers for Chinese jujube is urgently needed for use in genetic and breeding research.

SSR-enriched genomic libraries have been successfully applied in many plants, including Robusta coffee [26], Levant cotton [27] and peach [28]. The three-primer colony PCR screening strategy eliminates the need for colony hybridization to detect the desired inserts, resulting in substantial time and cost savings by minimizing the sequencing of inserts that do not contain the proper motif [23]. In this study, of the 2,030 sequenced positive colonies in the enriched libraries, 1,854 (91.3%) contained SSRs, similar to the results obtained from sunflower using a colony hybridization strategy (89%, [29]). This number is higher than those obtained for switchgrass (83.5%, [30]), eggplant (81.7%, [22]) and bunching onion (34.4%, [31]).

The percentage of unique clones in this study was 73.2%, higher than that reported in jute (67.3%, [32]), switchgrass (51.1%, [30]) and Italian ryegrass (25.6%, [33]). The proportion of redundant sequences was 19.7%, lower than those obtained in sorghum (33%, [34]) and chickpea (25.2%, [35]). Furthermore, the rate of successful amplification by the primer pairs (80.87%) obtained in this study is much higher than that obtained in eggplant (75.3%, [22]). These results indicate that the current enrichment procedure coupled with three-primer PCR screening efficiently generated a large number of SSR markers in Chinese jujube.

In this study, other types of motifs besides [AG]_n_(37.7%) and [AC] _n_(27.7%) were identified, including [AT]_n_, [CG]_n_, [AAC]_n_, [AAG]_n_, [AAT]_n_ and [ACC]_n_, possibly due to their abundance in the Chinese jujube genome [32]. AG-containing SSRs exhibited slightly higher development efficiency than AC-containing SSRs, in agreement with previous results for chickpea [35], zoysia grass [36] and timothy grass [37].

### SSR marker polymorphism

SSR markers exhibit much higher polymorphism than RFLPs, RAPDs, AFLPs and ISSR [38]. In the present study, the rate of polymorphic markers (86%) was higher than those obtained in sorghum (80.9%, [39]) and yarrow (53.3%, [40]). Botstein et al. [41] defined a locus with a PIC of 0.5 as highly polymorphic. In this study, 157 primer pairs met this criterion, providing an important tool for the evaluation of Chinese jujube genetic variability. We observed that markers derived from sequences containing dinucleotide repeats were generally more polymorphic than those containing trinucleotide repeats, in agreement with previous results for grape [42] and switchgrass [29]. Adjacent alleles are more easily separated and identified from one another using long nucleotide repeats compared to dinucleotide repeats[42]. In humans, long nucleotide repeats have been adopted for fingerprinting [43]. Studies of plant crops such as grape have also begun to employ SSR markers with long nucleotide repeats for fingerprinting [42]. The present study provided 35 SSR markers with repeats of three or more nucleotides, which will be valuable for use in the construction of fingerprints for Chinese jujube germplasm.

The level of polymorphism of an SSR is thought to be related to the number of repeats, as observed in *Pinus radiata* [44], eggplant [45] and peach [28]. However, we observed a very weak correlation (r = 0.12) between SSR length and PIC value, consistent with the results obtained in olive tree [46], bean [47] and *Cucumis* [38]. Thus, selecting loci with a sufficient number of repeats is not necessary to ensure the detection of higher polymorphism in Chinese jujube.

### Genetic diversity analysis of the main Chinese jujube cultivars

Scores of cultivars have been employed in the production of Chinese jujube; however, the level of genetic diversity has not yet been evaluated. Therefore, 31 primers with high PIC scores were selected to determine the level of genetic diversity among the major cultivars of Chinese jujube. All 76 Chinese jujube cultivars were uniquely identified, demonstrating a high efficiency of the primers in differentiating the cultivars.

In SSR data analysis, loci exhibiting two bands were scored as heterozygous at a single locus. A recent comprehensive study by Barthe et al. [48] confirmed the complex origin of genetic variation in the size and sequences of amplified microsatellites. If the observed bands correspond to duplicated DNA amplifications instead of variants of the same locus, the observed heterozygosity (Ho) and the expected heterozygosity (He) may be overestimated. Eighteen of the SSRs employed in this study were confirmed to follow Mendelian segregation (Pang et al., unpublished data), ruling out the possibility of duplicate loci. The population genetic parameters and structure obtained using these 18 SSR loci were similar to those obtained using 31 SSR loci; for this reason, we have reported the results obtained using the entire data set. The average values of the allele number (*Na*), the effective allele number (*Ne*), Ho and He were 5.7, 3.148, 0.678 and 0.621, respectively, which are higher than those obtained in other horticultural plant species, including apple [49], peach [28] and tomato. With the exception of BFU0277, BFU0249, BFU0478, BFU0479 and BFU0614, the fixation indices for the 31 primers were significantly less than zero, indicating an excess of heterozygotes. Moreover, the high average number of alleles amplified per locus (5.7) and the average observed heterozygosity values of 0.678 suggest that SSR diversity is comparatively high within Chinese jujube.

Cultivars from all seven populations were scattered among the three groups, and no population formed a distinct group in the dendrogram. These populations were defined on the basis of geography and thus might not reflect underlying genetic relationships. Most bootstrap values were less than 50% in the NJ clustering dendrogram, indicating that Chinese jujube has a complex genetic background resulting from frequent cultivar exchange among different areas and possible natural hybridization. Similar weak bootstraps have been reported for avocado (*Persea americana* Mill.) [50] and tree peony (*Paeonia suffruticosa* Andrews) [51]; these low values were hypothesized to result from the large number of hybrid genotypes in the data set and possible recombination among cultivar groups, respectively. This hypothesis is further supported by the observation of a slight excess of heterozygotes. These results suggest that the recorded location distribution of many Chinese jujube cultivars may not represent their real origin.

From the NJ dendrogram, it is apparent that ‘Changjixinzao’ and ‘Pingshunjunzao,’ ‘Dongzao100’ and ‘Dongzao70,’ ‘Xiangzao 45’ and ‘Xiangzao10,’ ‘Muzaokanglie’ and ‘Zhongyangmuzao,’ and ‘Wuhezao’ and ‘Jinsixiaozao’ have high genetic similarity, indicating a close genetic relationship. In a previous study, ‘Damaya’ and ‘Huluchanghong’ could not be differentiated by 113 SRAP fragments [11]. In the present study, 12 of the 31 SSR markers could be used to distinguish the two cultivars, demonstrating the differential power of the markers. ‘Wuhezao’ is possibly a sport from ‘Jisixiaozao’; only two differences (BFU0561 and BFU0308) were observed between these cultivars. The clustering results confirmed the close relationship between these cultivars, with a high bootstrap value of 83%. ‘Muzaokanglie’ has been described as a cultivar selected from ‘Zhongyangmuzao’ [52]; we observed differences in twelve SSRs between these two cultivars. Notably, several cultivars with similar cultivar names (which were expected to indicate a similar origin of the cultivars) did not cluster together in the dendrogram, including ‘Maya,’ ‘Damaya’ and ‘Beijingmaya,’ ‘Huizao3’ and ‘Huizao154,’ and ‘Changjixinzao’ and ‘Changjixinzao10.’. The cultivars that have the same usage do not cluster together, consistent with a previous result obtained using SRAP markers [11]. Taken together, the results of this study indicate that the microsatellite markers we have developed for Chinese jujube exhibit a high level of polymorphism, thus providing a powerful tool for genetic diversity studies and cultivar identification in germplasm collections.

## Conclusions

We reported the development of 1,188 SSR primer pairs from six enriched genomic SSR libraries of Chinese jujube. A set of 301 highly polymorphic SSRs was obtained using six Chinese jujube cultivars, 31 of which were employed to reveal a high level of genetic diversity among the major cultivars. The large-scale SSR markers developed here, together with their polymorphic information, represented a significant improvement in the available Chinese jujube genomic resources. These markers and their polymorphic information will be beneficial for both genetic and breeding applications to facilitate Chinese jujube improvement and accelerate the development of new cultivars.

## Materials and Methods

### Plant materials and genomic DNA isolation

All the plant materials were acquired with permissions from the National Key Base for Improved Chinese Jujube Cultivar, Cangzhou, China abiding by the laws in China. The plant materials used in this study did not involve endangered or protected species.

Fresh healthy leaves of 76 Chinese jujube cultivars were collected (Table 5). Z. *jujuba* ‘Dongzao’ was used for the construction of all genomic libraries. SSR primers were screened for polymorphism across a set of six cultivars that included ‘Dongzao’, ‘Muzao’, ‘Xiaoyazao’, ‘Lizao’, ‘Lingbaodazao’ and ‘Lajiaozao’, which were showed to be highly diverse previously [11]. Total genomic DNA was extracted from the leaves using a modified CTAB method [53]. DNA quality was measured by 1.0% agarose gel electrophoresis.

### Construction and sequence analysis of SSR-enriched genomic libraries

‘Dongzao’ was used to isolate microsatellites using magnetic bead enrichment as described in Nunome et al. [54]. Genomic DNA (20 µg) was digested with three blunt end-cutting restriction enzymes, *Alu* I, *Hae*III and *Rsa* I (Promega, Madison, Wisconsin, USA). The fragments then were ligated to a double-stranded linker (forward: 5′-GTT TAG CCT TGT AGC AGA AGC-3′; reverse: 5′-p GCT TCT GCT ACA AGG CTA AAC AAA A-3′) using T4 DNA ligase. The DNA fragments were hybridized with a 5′-biotin-(AG)_15_-3′ or 5′-biotin-(AC)_15_-3′ probe at 60°C overnight after incubation at 95°C for 15 min. The DNA hybridized to the probe was captured using streptavidin-coated magnetic beads (Promega, Madison, Wisconsin, USA) and washed three times in 0.2× SSC/0.1% SDS at room temperature. Finally, the DNA was eluted from the beads with 0.15 M NaOH. The eluted DNA fragments were amplified using DNA polymerase KOD-Plus (Promega, Madison, Wisconsin, USA), followed by adenylation of the 3′ ends of the PCR products. The adenylated PCR products were ligated to a pGEM-T-easy vector (Promega, Madison, Wisconsin, USA) and transformed into competent *Escherichia coli* DH5α (Promega, Madison, Wisconsin, USA). Recombinant colonies were identified using blue/white colony selection. The six enzyme and probe combinations resulted in six SSR-enriched genomic libraries designated as ALUAG, RSAAG, HAEAG, ALUAC, RSAAC and HAEAC. A three-primer (both forward and reverse vector primers and one synthesized complementary dinucleotide repeat primer without any degenerate nucleotides as an anchor) PCR-based procedure was used to screen microsatellite-containing colonies following the strategy reported by Wang et al. [23]. Positive clones were sequenced in one direction using an ABI PRISM 3730XL DNA sequencer (Applied Biosystems, Foster City, California, USA).

### Sequence checking and primer design

Sequences containing SSRs of 12 or more bases were identified using the MISA program (Microsatellite Identification Tool) from http://pgrc.ipk-gatersleben.de/misa. All sequences containing SSRs were analyzed for redundancy using the ClustalW program (http://www.genome.jp/tools/clustalw/). Only unique SSR clones with sufficiently long flanking sequences were used for primer design using the primer3 program (http://frodo.wi.mit.edu/). The sequences of the clones have been deposited to GenBank. The GenBank accession number for each clone sequence has been included in Table S1. All primers were designed using the following parameters: (1) product size from 100 to 350 bp; (2) primer size from 18 to 24 bp with an optimum size of 20 bp; (3) annealing temperature from 55 to 60°C with an optimum of 58°C; (4) GC content from 45 to 50%. An M13-tagged sequence (5′-TGT AAA ACG ACG GCC AGT-3′) was added to the 5′ end of the forward primer to enable detection with a universal fluorescently labeled M13 primer [55]. All primers were synthesized by GENEWIZ Biological Technology Co., Ltd. (Beijing, China).

### Polymerase chain reaction and fragment analysis

A third primer (M13F) labeled with a fluorescent (FAM, HEX, ROX, TAMRA) was used in the PCR reactions. PCR amplifications were performed using a GeneAmp PCR System 9700 thermal cycler (Applied Biosystems, Foster City, California, USA) in a 10- µl reaction volume containing 10–15 ng of template DNA, 2xTaq PCR mix (Biomed-Tech, Beijing, China), 1.6 pmol of each reverse and universal fluorescently labeled M13 primer and 0.4 pmol of the forward primer. The PCR amplification program was as follows: 94°C for 5 min; 30 cycles of 94°C for 30 s, 55°C for 40 s, and 72°C for 40 s; 8 cycles of 94°C for 30 s, 53°C for 40 s, and 72°C for 40 s; and a final extension at 72°C for 10 min [55]. The PCR products were subsequently detected using an ABI 3730XL DNA Analyzer and a GeneScan-500LIZ size standard (Applied Biosystems) and Gene-Marker software (SoftGenetics LLC, USA).

### Primer evaluation

To evaluate the efficiency of the microsatellites, 350 primer pairs exhibiting clear banding patterns were selected to evaluate polymorphism in six divergent cultivars. The number of alleles per locus and the PIC were calculated. PIC was calculated according to PIC = 1−∑P^2^_i_, where P_i_ is the frequency of the i^th^ allele among the total number of alleles in the sample [41].

### Analysis of genetic diversity

To determine the level of genetic diversity among the 76 Chinese cultivars, which were divided into seven populations according to their possible geographical orgin (Table 5). Thirty-one primers exhibiting high PIC values were selected for analysis. The amplification bands were corrected using FlexiBinv2 [56]. GenAlEx version 6.4, Microsatellite tools [57] and Cervus 3.0 were used to measure the variability in *Na*, *Ne*, Ho, He, Shannon's informative index (*F*) and PIC at each locus. The partition of observed genetic variation among and within populations and genetic groups was characterized using an analysis of molecular variance (AMOVA) as implemented in the program Arlequin version 3.5 [58].

### Genetic distance estimation and cluster analysis

Following the strategy employed by Federici et al.[59] and Pang et al. [60], SSR allelic data was transformed as present or absent (coded A or T, respectively) using DataTrans1.0 [61]. The genetic distance was calculated as the p-distance of nucleotide acids using MEGA 6.05, which is equivalent to that estimated using a simple matching coefficient, i.e., the proportion of shared A's and T's subtracted from 1. Furthermore, a neighbor-joining (NJ) dendrogram was constructed, and the robustness of the genetic relationships was evaluated using bootstrap analysis with 1,000 re-samplings using MEGA 6.05 [62].

## Supporting Information

Table S1 1188 SSR markers developed in the study. The detailed information including accession number for the sequence, SSR motif, repeat number, TM value, GC value, expected size and primer sequence for 1188 SSR markers developed in the study.
(XLS)

Table S2 The polymorphism of 350 selected SSR markers used in the study. The detailed information including SSR motif, repeat number and PIC value for 350 selected SSR markers used in the study.
(XLS)
